# Encoding fear intensity in human sweat

**DOI:** 10.1098/rstb.2019.0271

**Published:** 2020-04-20

**Authors:** Jasper H. B. de Groot, Peter A. Kirk, Jay A. Gottfried

**Affiliations:** 1 Department of Neurology, University of Pennsylvania, 3400 Spruce Street, Philadelphia, PA 19104, USA; 2 Department of Psychology, Utrecht University, Heidelberglaan 1, Utrecht 3584 CS, The Netherlands; 3 Department of Psychology, University of Pennsylvania, 3720 Walnut Street, Philadelphia, PA 19104, USA

**Keywords:** smell, fear, sweat, autonomic nervous system, multivariate pattern classification, photo-ionization detector

## Abstract

Humans, like other animals, have an excellent sense of smell that can serve social communication. Although ample research has shown that body odours can convey transient emotions like fear, these studies have exclusively treated emotions as *categorical*, neglecting the question whether emotion *quantity* can be expressed chemically. Using a unique combination of methods and techniques, we explored a dose–response function: Can *experienced* fear intensity be *encoded* in fear sweat? Specifically, fear experience was quantified using multivariate pattern classification (combining physiological data and subjective feelings with partial least-squares-discriminant analysis), whereas a photo-ionization detector quantified volatile molecules in sweat. Thirty-six male participants donated sweat while watching scary film clips and control (calming) film clips. Both traditional univariate and novel multivariate analysis (100% classification accuracy; *Q*^2^: 0.76; *R*^2^: 0.79) underlined effective fear induction. Using their regression-weighted scores, participants were assigned significantly above chance (83% > 33%) to fear intensity categories (low–medium–high). Notably, the high fear group (*n* = 12) produced higher doses of armpit sweat, and greater doses of fear sweat emitted more volatile molecules (*n* = 3). This study brings new evidence to show that fear intensity is encoded in sweat (dose–response function), opening a field that examines intensity coding and decoding of other chemically communicable states/traits.

This article is part of the Theo Murphy meeting issue ‘Olfactory communication in humans’.

## 1. Introduction

Humans use multiple sensory modalities to express themselves in a social world. An important element to everyday social interactions is the expression of our emotions, which can be conveyed through our face, body, speech, touch and even our smell. Among these modalities, the sense of smell is arguably the most underestimated one. Yet, empirical studies have confronted traditional ideas that human olfaction is inferior [1,2]. For instance, humans can use their noses to follow a scent [3]—in some instances we outperform super-smelling rodents by detecting certain odorous molecules at lower concentrations [4]—and humans can learn how to discriminate previously indistinguishable smells [5]. What we also share with other animals is an apparent capacity to use smells (body odour) for social communication purposes [6]. Various studies have shown that human body odours can express a person's identity, gender, age, health status and emotional state (recent reviews: [7,8]). Among the emotions that humans appear to convey by smell are anger/aggression [9], disgust [10] and happiness [11], but ‘the smell of fear’ has attracted the most attention (recent meta-analysis: [12]).

Whereas close to 30 studies have yielded perceptual, behavioural and neural evidence for the communication of fear via body odour [12], these and other studies have been limited by exclusively focusing on emotions as discrete categorical entities or *qualities* (e.g. fear, neutral, disgust), neglecting the question whether emotion intensity or *quantity* can be expressed in a person's body odour. Emotion intensity can be readily encoded in a person's face [13] and voice [14], which serves the important communicative function of affecting a decoder's behaviour; yet, the precise mechanisms of how intensity of emotion experience is chemically encoded in a person's body odour (or sweat) have yet to be unravelled. Building on a rich database of studies [12], the focus here is on answering that question for fear. Specifically, is there a ‘dose–response’ relationship between intensity of experienced fear and quantity of odorant molecules contained in fear sweat?

### (a) Experienced fear intensity

Before addressing the question whether fear intensity can be *encoded* in sweat, the first challenge is to characterize what a *fear experience* entails. Determining effective fear induction is crucial, especially in resource-intensive studies in the field of human chemical communication (if fear is not encoded, it cannot be decoded). To quantify emotion experience, the emotion literature in general has historically relied on univariate methods. Although univariate approaches could disrupt the continuity of subjective and physiological response patterns by parsing a Gestalt emotion experience into separate non-representative pieces [15], these analytical methods have exclusively been applied to determine experienced fear of the so-called donors. To induce fear, studies capitalized on the idea of emotional contagion, as might be induced by watching scary videos [16], skydiving for the first time [17], participating in a high rope course [18] or awaiting an important academic examination [19]. In these studies, successful fear induction was based on self-report questions (e.g. more reported fear, anxiety) typically combined with *one* physiological endpoint, including—in the order of frequency—higher heart rate (HR), more armpit sweat production, higher cortisol, higher skin conductance levels (SCL) and higher respiratory rate (RR) (see [12], for a list of studies). Occasionally, these variables produced inconsistent results. In a small study (*N* = 7) that recorded multiple physiological endpoints (analysed in univariate fashion), scary videos elicited higher RR, but not higher HR, SCL and armpit sweat quantity [16]. In fact, no physiological variable was consistently higher during the sampling of fear sweat (the same applies to self-reported fear: [10]), which underlines the insensitivity of a univariate approach in determining fear experience, which produces a serious yet unnoticed challenge.

A promising alternative is provided by multivariate techniques that can quantify and classify emotion experience based on a greater receptivity for coordinated autonomic nervous system (ANS) activity and subjective emotion experience (e.g. [15,20]). Such an approach of capturing the whole emotion experience is compatible with both a functional view [21,22] and ‘natural kinds’ view (e.g. [23,24]) of basic emotions as coordinated response systems. Multivariate classification of emotion experience is not widespread; yet, when applied in the context of emotion research, it has provided strong and replicable support for the (autonomic) specificity of basic emotions like fear (e.g. [15,20]). In those studies, emotions were induced using videos, a well-validated technique that has been applied before in the context of sweat sampling research (for reviews, see [7,8]), and which will be used again here to induce fear to collect fear sweat.

In the present study, participants' fear experience was quantified in multivariate fashion using a novel application of the multivariate analysis technique partial least-squares-discriminant analysis (PLS-DA), which is recommended over regular discriminant analysis (DA) when variables are moderately correlated (as anticipated here). Specifically, we introduce PLS-DA (i) to quantify the success of separating fear responses from neutral responses; (ii) to quantify the relative contribution of each subjective and physiological variable to this classification process; and (iii) to produce a weighted regression equation through which participants could be assigned to categories of quantified experienced fear intensity. As such, PLS-DA could provide a novel and thorough perspective on what a fear experience entails, and how intensely the different subjects experienced fear, before taking the next step by addressing the question whether the strength of fear experience could be expressed in (armpit) sweat.

### (b) Encoding fear intensity in sweat

Another quantification query exists concerning the molecules embedded in the fear sweat. Even though sweat is a dynamic stimulus that ostensibly degrades over time owing to skin bacteria metabolizing the sample on the medium (sweat pad) to which they are transferred [8], there has been virtually no attempt to quantify the influence of certain methodologies (from emotion induction to post-collection storage) on the sweat stimulus. The only exception has been a pioneering study by Lenochova *et al*. [25], which showed that perceptual ratings of body odour samples (e.g. intensity, pleasantness) remained stable after repeated freeze–thaw cycles (up to six months). However, the absence of evidence for rating differences does not entail evidence of absence, and human biases including training effects cannot be ruled out, calling for additional objective indicators of sweat.

Supplementing prior research [25], we explored and quantified the ‘physical ground truth’ of sweat in terms of the number of molecules that are emitted (quantity of chemical composition), and how volatile quantity evolves over time (stimulus constancy versus decay). We used a photo-ionization detector (PID), which objectively detects the quantity of molecular volatiles. Aside from charting stimulus consistency over time, the main question entails whether a higher dose of fear sweat is linked to more volatile molecules being emitted by the sweat (dose–response relation).

### (c) Hypotheses

The present research entails a novel combination of a multivariate classification technique (PLS-DA) and PID measurements to answer the question whether the intensity of a fear experience can be encoded in sweat. Buttressed by prior research, we hypothesized a dose–response relationship between fear intensity experienced by encoders (quantified with PLS-DA) and fear intensity encoded/expressed in sweat (quantified with PID recordings). Specifically, we made the following predictions: (i) using scary film clips, participants would experience discrete fear (beyond high negative arousal *per se*); (ii) capitalizing on individual response variability, participants' fear experience will be classified into different intensities (low, medium and high); (iii) stronger fear experiences will translate into more produced (armpit) sweat; and (iv), higher doses of fear sweat will be linked to more volatile molecules being emitted by the sweat, providing decoders with a quantity cue/signal.

## 2. Methods

The University of Pennsylvania's Institutional Review Board approved this research (Protocol no. 828758). Informed consent was obtained from all subjects.

### (a) Experiment 1a: sweat sampling

#### (i) Participants and design

Forty healthy male subjects (age: mean = 22.67 years, range = 18–38) ‘donated’ armpit sweat while watching fear clips and a calm-neutral video (control condition) in two counterbalanced 30 min sessions (within-subjects) separated by a 10 min break.

Inclusion criteria were based on prior protocols (e.g. [10,26]): only males were recruited, as they have larger and more active apocrine sweat glands in their armpits [27], which are linked to fear sweat production [28], thus increasing the study's effectiveness. Participants were also screened for significant neurological or psychiatric diseases, chronic medical illness, cardiac conditions, alcoholism, smoking status (less than six months previously), consistent drug use, and non-fluency in English. Online screening further prevented the inclusion of subjects that found horror movies *not* scary (for the experiment's sake) or *too* scary (‘I cannot watch it; I will walk away …’; for the participants' sake).

Despite passing the initial screening, one participant was excluded owing to his extremely high calmness scores (*Z* = 5.85) and low fear scores (*Z* = −2.13) in the fear condition. Three participants were excluded owing to missing skin conductance data (technical error), leaving 36 subjects for the final analyses.

#### (ii) Materials and measures

*Emotion induction.* Being easy to implement, movie clips are among the most popular and potent triggers of emotions in a laboratory setting (meta-analysis: [29]), evoking changes in both subjective experience and physiology (e.g. [30]). Prior fear sweat sampling research has relied repeatedly on fear-inducing clips [12] drawn from a large pre-validated database [31]; however, as this database has ‘aged’ and their familiarity (e.g. ‘The Shining’) could have reduced clips' effectiveness, the current selection of fear-inducing clips was based on a pilot test (*N* = 15) including several recent clips (see electronic supplementary material, figure S1). As wildlife documentaries induce a calm ‘control’ state [32], the neutral condition consisted of parts from BBC's documentary ‘Yellowstone: Autumn’ (used before in, e.g., [33]).

*Sweat production.* Armpit sweat was sampled on 4 × 4 inch sterile absorbent compresses (Curad Pro-Gauze, Medline Industries, no. 080196305226) placed under the left and right armpits. To determine sweat production each ‘sweat pad’ was weighed (0.01 g precision) pre- and post-emotion induction. Total sweat production was the summed weight increase of the left and right sweat pads.

*Galvanic skin response.* Fear and anxiety increase skin conductivity following sweat gland activity, known as the GSR. The GSR complex includes a slower-acting *tonic* component (SCL) and a rapid *phasic* component (skin conductance responses: SCR), which are linked to separate (neural) mechanisms [34]. GSR was measured on the left hand, using bipolar Ag–AgCl GSR Finger Electrodes (MLT117F, ADInstruments, USA) that were attached to the medial phalanx of the index finger and ring finger (preventing electrode contact artefacts). To improve skin contact and recording quality, participants' fingers were cleaned with water (involving no soap/alcohol [35]) and dried, while electrodes were filled with gel containing 0.5% saline in a neutral base (TD-246, Florida Research Instruments, USA). The GSR signal was amplified (GSR Amp FE116, ADInstruments) and calibrated (zeroed) in two stages: before connecting the electrodes to subjects (open circuit zero), and afterwards (subject zero), following a recommended 2 min baseline [34].

*Heart rate.* HR was recorded using an infrared photoelectric sensor (plethysmograph; MLT1020PPG, ADInstruments) that was Velcro-strapped to the left thumb to detect beat-to-beat changes in pulsatile blood flow.

*Respiratory rate.* Changes in respiration were recorded using an unobtrusive breathing belt (TSD221-MRI, BIOPAC Systems, USA), which contained a respiration sensor in a mesh strap with self-adhering band (70 cm) that was affixed to the subject's abdomen (tight, but not uncomfortable). Respiration signals were transduced by a differential pressure transducer (ML311 Spirometer Pod, ADInstruments).

*Emotion questionnaire.* Four questions about discrete emotions (surprise, anger, fear and disgust) supplemented 16 items (e.g. happy, sad, nervous, fatigued) from Russell's circumplex model [36], which maps emotions in two-dimensional space containing arousal and valence dimensions. Participants first rated the presence/absence of a randomly presented state. Answering ‘no’ meant their score was 0. Answering ‘yes’ meant they additionally rated intensity on a 5-point scale from 1 (a little bit) to 5 (extreme). Experienced arousal and valence were also assessed directly [37].

#### (iii) Procedure

Participants followed a pre-validated protocol (e.g. [8,10,26,33]) to avoid sweat ‘contamination’ starting 48 h pre-donation. First, participants shaved their armpits to improve sweat collection. Throughout the pre-donation period, the consumption of heavily flavoured food items, alcohol and drugs, excessive physical exercise and sexual activity were prohibited. Donors were provided with scent-free hygiene products to use in the pre-donation period (e.g. fragrance-free deodorant containing potassium alum), and they filled in a diet diary to monitor consumption behaviour. On the donation day, donors wore a clean T-shirt to prevent odour contamination from their clothes. The experimenter first checked participants' compliance to the study's restrictions (in case of violations, participants were rescheduled). The participant then entered a room (temperature: 23.7°C, relative humidity: 22.4%) where sweat collection would take place. Before each sweat collection session, participants thoroughly cleaned and dried their armpits with water wipes and unbleached paper towels (monitored by an experimenter), while a second experimenter (wearing nitrile gloves) weighed the absorbent pads before attaching them to donors' armpits using hypoallergenic tape. Participants put on a new clean T-shirt and hooded sweater and were seated in front of a computer, while physiological recording instruments were applied. Participants were asked to keep still during the experiment to avoid movement artefacts. To establish a recommended 2 min baseline for physiological recordings, participants first viewed a relaxing beach scene, after which GSR and respiratory signals were zeroed. Then, participants saw either the 30 min fear-inducing clips or a wildlife documentary (counterbalanced), directly followed by the emotion questionnaire. Stimulus presentation and questionnaire data collection were controlled by a laptop running Inquisit 5.0 Millisecond Software. On another laptop, physiological signals were continuously recorded (1000 Hz) using a Powerlab 8/35 data acquisition system and LabChart8 software (ADInstruments). Finally, the experimenter removed the recording instruments and sweat pads, which were weighed and frozen in pre-coded 100 ml polypropylene jars (−80°C) for subsequent experiments.

#### (iv) Statistical analysis

*Data pre-processing.* Physiological data analysis was performed after band-pass filtering raw signals (HR: 0.5–50 Hz; RR: 0.05–50 Hz; phasic SCR: 0.05–35 Hz) to remove drift and artefacts. LabChart8's pre-set functions were used to detect heartbeat frequency (settings: ‘Cardiovascular–Finger Pulse’), breath frequency (settings: ‘Respiration–Respiratory Belt’), and SCR frequency (simple threshold: 0.03 µS; cf. [34]). Raw mean SCL (μS) reflected changes from a subject's baseline (0 µS).

*Univariate analysis.* Data (permanently stored on: https://osf.io/4dtqb/) were first subjected to traditional univariate analysis to chart effective fear induction (versus neutral control) on each *separate* physiological and subjective endpoint (e.g. [11,26]). Non-parametric tests and boxplots were reported, because data generally were not normally distributed.

*Multivariate analysis.* A multivariate approach towards determining effective fear induction was conducted using PLS-DA in XLSTAT software (Addinsoft, USA). PLS-DA calculated general classification accuracy (% donors correctly classified into the fear or neutral condition). Each variable's share in this classification process was quantified and expressed in a weighted regression equation, with larger weights belonging to better performing variables. Multiplying these regression weights with participants' raw scores (obtained in the fear condition) resulted in a *composite fear score* (CFS). Using the CFS ranking, the 36 donors were classified in fear intensity groups. Classification adequacy was assessed via leave-one-out cross-validation (LOO-CV), which involved sequentially omitting each participant's response, and subsequently repeating the discriminant analysis (DA) to verify classification accuracy of the omitted case. A binomial test was then used to check whether donors were classified above chance proportion (one-third) into the low, medium and high fear categories.

**Figure 1.**
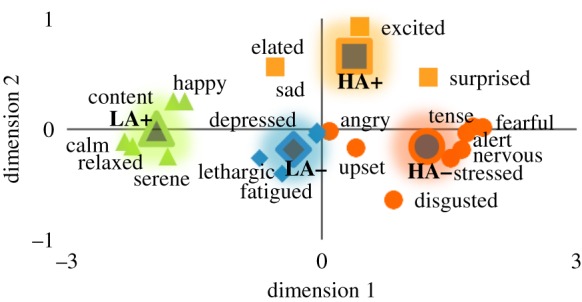
Clustering of self-reported feelings in quadrants of arousal (high versus low) and affect (+ versus −). Triangles, LA+ items; diamonds, LA− items; squares, HA+ items; circles, HA− items; dark grey shapes, centre of each cluster. (Online version in colour.)

*PLS-DA assumptions.* PLS-DA was chosen over regular DA, because PLS-DA has better tolerance to the moderately correlated (*r* ≈ 0.5) predictor variables found in the present study. Still, the problem that highly correlated (*r* > 0.7) self-report variables (e.g. fearful, nervous and tense) would introduce substantial multicollinearity and concomitant instability of regression coefficients was averted, by combining rating questions into separate, relatively uncorrelated clusters. There exist precedents to cluster feelings into four combinations of arousal (high, low) and valence (positive and negative affect; e.g. [36,38]). To corroborate whether questionnaire data fitted into these quadrants, multidimensional scaling was applied with ALSCAL (IBM SPSS Statistics 25), which created a two-dimensional Euclidean distance model using a least-squares algorithm. Items that correlated best with other items around a group centroid were clustered into that respective group. This approach resulted in four categories (figure 1): high arousal–positive affect (HA+: surprised, excited, elated); high arousal–negative affect (HA−: upset, stressed, nervous, tense, alert, fearful, angry, disgusted); low arousal–negative affect (LA−: sad, depressed, lethargic, fatigued); and low arousal–positive affect (LA+: happy, content, serene, relaxed, calm).

When the new arousal–valence clusters (HA+, HA−, LA+, LA+) were entered in PLS-DA with the remaining arousal and valence item, the latter items (arousal, valence) introduced substantial multicollinearity (variable inflation factor: VIF = 6.26); their removal caused VIFs to return to acceptable levels (less than or equal to 3.92). Furthermore, because extreme cases would cause underweighting in PLS-DA, outliers were identified using the most robust measure in the presence of outliers, the median absolute deviation (MAD) [39], and scores exceeding 2.5 MAD units were Winsorized (fear cases: 3.03%; neutral cases: 2.78%) [40].

### (b) Experiment 1b: sweat quantification

#### (i) Samples

Sweat samples were divided into three categories to explore different questions: (i) fear sweat (156 mg/75 cm^2^) versus neutral sweat (28 mg/75 cm^2^); (ii) higher fear intensity sweat (113 mg/75 cm^2^) versus lower fear intensity sweat (45 mg/75 cm^2^), and (iii); first time used versus second time used sweat. All samples used for Experiment 1b came from three donors (the remaining samples were used in an extensive ‘decoder study’, reported elsewhere, which measured decoders' behavioural, neural and psychophysiological responses to the sweat samples). The three donors were representative of the larger male donor sample (*N* = 36) in terms of age (mean = 25.25, range 20–35), their adherence to a scent-free protocol, and amount of sweat produced in the lower fear intensity condition (45 mg versus 48 mg for donors classified in low and medium fear intensity), higher fear intensity condition (113 mg versus 128 mg for medium–high fear donors) and neutral condition (28 mg versus 36 mg, *N* = 36). Whereas the higher and lower fear intensity sweat stemmed from single donors, the fear and neutral sweat (used twice) were pooled across the three donors and armpits, following typical procedures [8]. There were no significant differences in sweat production between the left and right armpit, *Z* values < 1 (whole sample: *N* = 36).

#### (ii) Photo-ionization detector

A PID (200B miniPID, Aurora Scientific, Canada) quantified the number of volatiles given off by the sweat samples. The highly sensitive PID sensor with a 2.25 inch inlet needle drew in air through a suction pump, after which molecules with ionization energy < 10.6 eV were ionized by high-intensity ultraviolet light. This meant that natural constituents of air (e.g. oxygen) were not detected, while the PID did detect sweat molecules (e.g. acetic acid). Ionization caused a current proportional to the molecules’ concentration. The PID sensor head was connected to a miniPID controller (gain: × 5; pump: high). Recordings started when the PID signal was zeroed in LabChart8 after a recommended 30 min warm-up period.

#### (iii) Olfactometer

A four-channel, computer-controlled olfactometer delivered sweat stimuli to the PID, which recorded *N* sweat volatiles through an inlet needle inserted approximately 1 inch into the olfactometer tube endpiece. First, sweat stimuli (75 cm^2^ of fear, neutral pads) with different weights were randomly distributed over four wide-mouth amber bottles (60 ml, Fisherbrand, USA). The bottles' polyvinyl-lined caps contained holes for Versilon Inert Tubing (SE-200, 1/8 inch inner diameter, 1/4 inch outer diameter) (US Plastic, USA), such that medical-grade room air could ‘transport’ the sweat stimulus to the endpiece. A Matlab script (Mathworks, USA) would trigger the randomized opening of one of four odour valves and its connected odour channel (*mean* inter-trial time: 12.5 s). The olfactometer was equipped with two independent mass flow controllers (Alicat Scientific, USA) that kept a constant air flow (3.2 l min^−1^). During stimulus presentation, 95% of air (3.04 l min^−1^) travelled over the sweat pad (odour channel), while 5% followed a separate ‘air channel’, devised to washout residual odour in-between stimulus presentations. A 60 min (pilot-tested) thawing time was maintained prior to any recording; each recording session lasted approximately 60 min. First time used sweat samples were refrozen immediately after a recording session and, prior to the second session, were thawed once more (60 min).

## 3. Results

To answer the question of whether fear intensity is encoded in sweat, a series of co-dependent questions were addressed: (1) Can *discrete* fear be induced in donors? (2) If so, can donors' fear experience be classified above chance in different intensities (low, medium, high)? (3) Does a stronger fear experience translate into more produced sweat? (4) Is more sweat linked to more volatile molecules being emitted by the sweat?

### (a) Induction of discrete fear experience (Hypothesis I)

#### (i) Univariate approach

**Figure 2.**
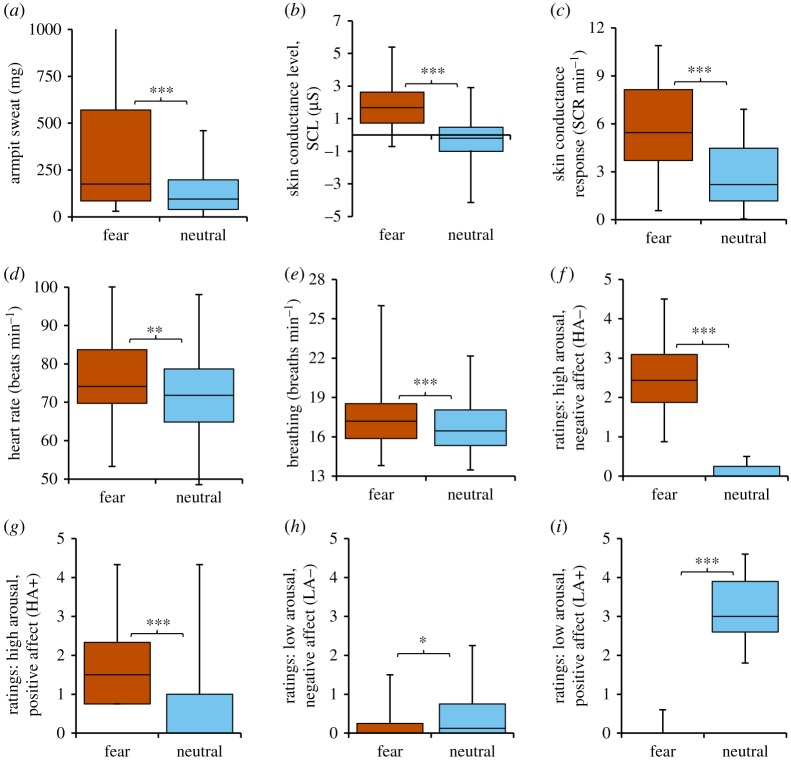
Univariate results showing effective fear induction on physiological and subjective measures. Boxplots display the distribution of the data, their full range from minimum to maximum (whiskers), the 25–75% range (box), and the median (middle of the box). Significance: *p < 0.05, **p < 0.01, ***p < 0.001. (Online version in colour.)

First, a traditional univariate approach was conducted to determine effective fear induction through separate analysis of physiological indicators of fear and self-report measures. Compared with the calm-neutral condition, fear-induced donors (*N* = 36) produced more armpit sweat (figure 2), greater galvanic skin responses (SCL, SCR), higher RR (all *p* values < 0.001), and higher HR (*p* = 0.004). On subjective indicators, the fear condition evoked the highest scores on high arousal–negative affect (HA−) items (*p* values < 0.001). Specifically, donors induced to be fearful actually felt more fearful, tense, nervous and alert, compared with non-targeted HA− states: angry, upset and disgusted (*p* values < 0.001) (see electronic supplementary material, table S1, for more details).

#### (ii) Multivariate approach

**Figure 3.**
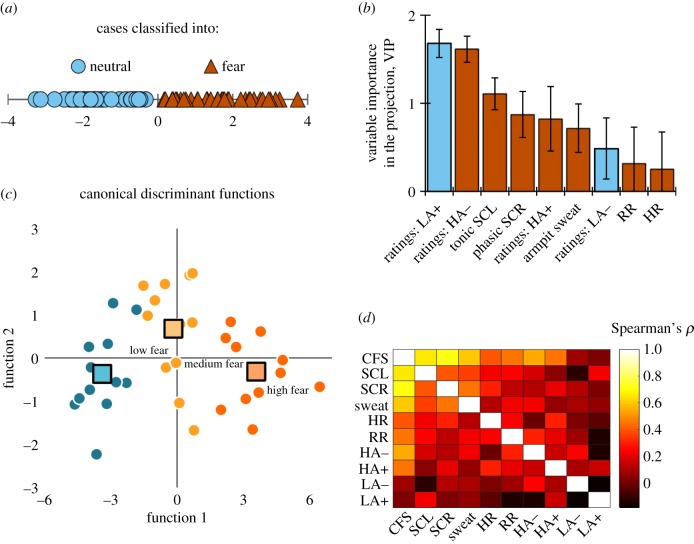
Multivariate classification of fear responses. (*a*) 100% correct classification of donors into fear and neutral. (*b*) Explanatory variables contributing most to the model based on ‘variable importance for the projection’ (VIP) scores (95% confidence intervals). Brown bars: higher values mean a greater likelihood of fear response (versus blue: neutral). (*c*) Classification of fear-induced donors into fear intensity categories. (*d*) Heat map of correlations between composite fear score (CFS) and physiological and subjective measures. SCL, skin conductance level; SCR, skin conductance response; HR, heart rate; RR, respiratory rate; HA−, high arousal–negative valence questions; HA+, high arousal–positive valence questions; LA−, low arousal–negative valence questions; LA+, high arousal–positive valence questions.

The multivariate technique PLS-DA was then used to separate classes of fear responses and neutral responses. The multivariate model (goodness of prediction: *Q*^2^ = 0.76, *R*^2^ = 0.79) separated donors’ fear responses from their neutral responses with 100% classification accuracy (figure 3*a*), based on subjective ratings (clusters: HA−, HA+, LA−, LA+) and physiological responses (SCL, SCR, armpit sweat, HR, RR).

### (b) Categorization of fear experience intensity (Hypothesis II)

Before assigning donors (*N* = 36) to different categories of fear intensity, PLS-DA's classification function was used to produce a regression equation that weighted (using unstandardized regression coefficients) the unique contribution of each subjective and physiological variable to yield the best classification of fear (versus neutral) responses (for an absolute ranking, see figure 3*b*; for details, see the electronic supplementary material). Subsequently, a CFS was computed per subject by multiplying each weight by participants' raw score during fear induction:

$$\begin{array}{rl} \text{CFS}= & -0.096+0.053\;\times \;(\text{tonic }\;\text{SCL})+\;0.024\;\times \\ & \;\;(\text{phasic}\;\text{SCR})+\;0.002\;\times \;(\text{HR})+0.012\;\times \\ & \;\;(\text{RR})+\;0.348\;\times \;(\text{sweat}\;\text{ pad}\;\text{weight})+\;0.095\;\times \\ & \;\;(\text{ratings}:\;\text{HA}-)\;+\;0.060\;\times \;(\text{ratings}:\;\text{HA}+)\;-\;0.071\times \\ & \;\;(\text{ratings}:\;\text{LA}-)\;-\;0.080\;\;\times \;(\text{ratings}:\;\text{LA}+). \end{array}$$

Subjects’ CFSs were then used to subdivide fear-induced donors into three groups (*n* = 12) of experienced fear intensity, namely low (*mean* = 0.63, s.d. = 0.06), medium (mean = 0.88, s.d. = 0.06) and high (mean = 1.17, s.d. = 0.10). Leave-one-out cross-validation (LOO-CV) corroborated in unbiased fashion that donors' fear experience was appropriately categorized into fear intensity categories: 30 out of 36 donors (83.33%) were correctly classified into categories of low fear, medium fear and high fear (figure 3*c*). The strength of the findings was underlined with a binomial test showing significant above-chance (33.33%) classification of donors into ‘low fear’ (91.67%), exact binomial *p* (one-tailed) < 0.001, ‘medium fear’ (75.00%), *p* = 0.004 and ‘high fear’ (83.33%), *p* < 0.001.

### (c) Fear intensity and (armpit) sweat dose (Hypothesis III)

The next, more specific, question was whether a stronger fear experience would translate into more (armpit) sweat production. To test this, a one-way ANOVA was conducted on produced armpit sweat with fear intensity category (low, medium, high) as factor, which indicated a significant linear contrast effect, *F*_1,35_ = 24.85, *p* < 0.001, *η*^2^ = 0.42. As expected, armpit sweat production increased from the low fear group (*n* = 12: mean = 47.19 mg/75 cm^2^, s.d. = 20.13 mg), to the medium fear group (*n* = 12: mean = 77.19 mg/75 cm^2^, s.d. = 68.67 mg), to the high fear group (*n* = 12: mean = 160.31 mg/75 cm^2^, s.d. = 64.42 mg). Aside from categorical differences in armpit sweat production from low to medium to high, there were also linear differences: a stronger fear experience, as indicated by participants' (*N* = 36) CFS, was strongly correlated with armpit sweat production, Spearman's *r*_35_ = 0.60, *p* < 0.001.

Armpit sweat production was also significantly correlated with galvanic skin responses (SCR: *r*_36_ = 0.45, *p* = 0.006; SCL, *r*_35_ = 0.36, *p* = 0.030; see figure 3*d*, for a full correlation heat map). Whereas SCR and SCL are a product of *eccrine* sweat gland activity [35], fear sweat production has been linked to *apocrine* sweat gland activity [28], and the armpit contains both gland types [8]. Notably, eccrine glands produce odourless water and electrolytes, while the apocrine glands excrete initially odourless precursor molecules with a potential to become volatile [8]. Knowing this, the next open query was examined, namely whether armpit sweat produces volatile molecules (versus odourless secretion), and whether fear intensity and concomitant larger quantities of armpit sweat were expressed in *more* volatile molecules being emitted.

### (d) Armpit sweat and volatility: dose–response and decay (Hypothesis IV)

**Figure 4.**
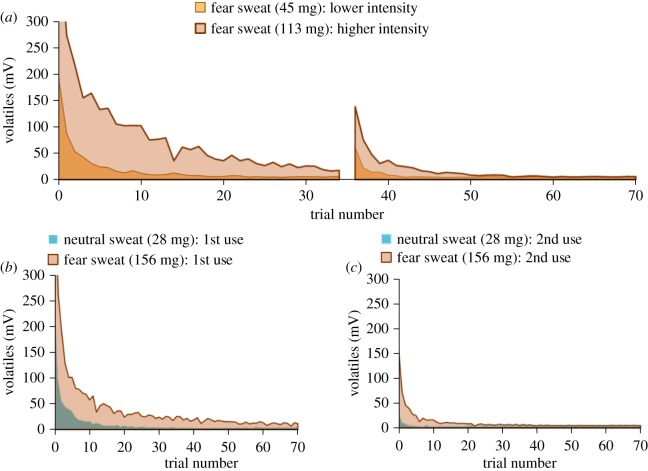
Quantity of volatiles emitted by sweat (expressed in mV) as a function of time, pad weight and emotion. (*a*) Higher intensity fear sweat emits more volatiles than lower intensity fear sweat. A mid-way break caused volatiles to build up in the headspace, producing temporarily higher values. (*b*) Fear sweat emits more volatiles than neutral sweat. (*c*) Reused sweat (second use) emits fewer volatiles than first time used sweat (first use). (Online version in colour.)

A PID quantified whether more armpit sweat as a function of fear degree could be linked to a higher quantity of volatile molecules being emitted, with PID current being proportional to molecule quantity. Despite the small yet representative sweat samples from three male donors (see Methods), volatile quantity was explored over six sweat samples (i.e. one ‘lower intensity fear’ sample from one individual, one ‘higher intensity fear’ sample from another individual, and a fear and neutral sweat sample pooled over three individuals tested twice: first time use versus second time use) to determine how their properties changed over a 30 min time window (35 trials), and predictable patterns emerged (figure 4).

A multiple linear regression model was able to predict quantity of volatiles (expressed in millivolts, mV) emitted by sweat on the first exposure (i.e. a critical time-point focused on in many previous fear odour studies: [12]), based on sweat pad weight (*p* = 0.019), emotion (fear versus neutral: *p* = 0.040) and pad freshness (first use versus second use: *p* = 0.017), *F*_3,5_ = 29.35, *p* = 0.033, with an *R*^2^ of 0.98. The relatively high number of volatiles emitted at the start of the experiment was arguably caused by volatiles assembling in the vial's headspace prior to release. Although sweat stimuli decayed over time (see below), subsequent exposures still yielded regression equations with high variance explained (*R*^2^_mean_ = 0.86; all *R*^2^ values > 0.70).

Three consistent patterns emerged in the data (figure 4). First, sweat pads with *higher weight* emitted more volatiles. To illustrate, two different *types of fear sweat*, one at a lower weight (43 mg) and one at a higher weight (113 mg) yielded marked differences in volatile quantity, with lower fear intensity sweat giving off fewer volatiles (median = 5.60 mV, interquartile range (IQR) = 5.00–9.40) than higher fear intensity sweat (median = 24.70 mV, IQR = 6.65–58.15). Second, fear sweat (median = 10.33 mV, IQR = 7.61–16.90) emitted more volatiles than neutral sweat (median = 5.08 mV; IQR = 4.85–5.86), regardless of stimulus freshness. Third, first time used sweat emitted more volatiles (median = 10.10, IQR = 7.50–16.89) than second time used sweat (Mdn = 5.23 mV, IQR = 5.00–6.55), irrespective of emotion (fear sweat: 156 mg, neutral sweat: 28 mg).

General sweat pad ‘decay’ (fewer emitted volatiles) over multiple exposures was underlined by a repeated measures ANCOVA with time as within-subjects factor, and covariates sweat pad weight, pad freshness (first use versus second use), and induced emotion (fear versus neutral), *F*_34,68_ = 2.23, *p* = 0.003, $\eta_{p}^{2}=0.53$. Curve fitting in Matlab indicated that all sweat pads showed a clear bi-exponential decay function in terms of their volatility (all *R*^2^ ≥ 0.95), with an initial sharper volatile quantity drop followed by slower decay. These bi-exponential functions outperformed all other functions, including simple exponential fits (average *R*^2^ = 0.73).

## 4. Discussion

Our main goal was to explore whether fear intensity could be encoded in human body odour. The question whether emotion quantity can be expressed in a person's sweat had been neglected amidst comparable studies that exclusively focused on the chemical transfer of qualitatively different emotions (e.g. fear, disgust). Here, multivariate statistical analysis (PLS-DA) was used to quantify fear experience, while a PID quantified volatiles emitted by sweat. These methodologies had never been applied to the field of human chemical communication; yet, through this unique combination, we could identify a dose–response function between experienced fear intensity and intensity encoding in fear sweat.

The novel multivariate approach (100% classification accuracy) and traditional univariate analysis of physiological and subjective endpoints converged by showing effective fear induction using scary videos. Compared with the calm-neutral condition, fear responses were characterized by more produced armpit sweat (e.g. [11,33]), higher respiratory rates [16], more galvanic skin responses and higher HR (e.g. [41]). Subjectively, the fear condition evoked higher scores on high arousal–negative affect items (e.g. feeling more fearful, tense and nervous), and lower scores on low arousal–positive affect items (e.g. calmness, serene). Another function of the multivariate approach was a weighted regression equation produced to determine ‘fear group membership’, which helped assign participants to categories of low, medium and high experienced fear. Our classification of fear intensity was significantly above chance (83% versus 33%), as indicated by unbiased leave-one-out cross-validation and binomial tests. Our models furthermore showed that participants experiencing more fear also produced more armpit sweat, and PID recordings revealed that greater quantities of fear sweat meant more volatile molecules were contained in the sweat, pointing to a dose–response function between fear intensity *experience* and *encoding* (in sweat).

The obtained insights regarding fear intensity encoding in sweat were limited to male sweat samples, which forms a constraint on generality [42]. Currently, we do not have evidence that our findings regarding intensity encoding generalize to females. Only males were recruited for this proof-of-principle study to enhance this study's effectiveness, because males have larger and more active apocrine sweat glands in their armpits [27], which are linked to fear sweat production [28]. However, we have no reason to believe that females *cannot* produce fear sweat at all, as prior research focusing on qualitative differences (fear versus neutral) showed no difference in fear-inducing effects of male and female fear sweat [43], and a meta-analysis showed that one-third of the used fear sweat samples came from female donors [12]. We believe that, at most, the smaller and less active apocrine sweat glands in females may affect models separating fear intensity groups through potentially lower effect sizes.

Despite this generality issue, robust results emerged using the male sweat samples, including the serendipitous finding that over multiple trials and uses, the sweat samples degraded consistently (i.e. emitted fewer volatiles). The present results seem at odds with earlier findings that repeated freeze–thaw cycles did not influence hedonic ratings of sweat [25]; yet, the ways of presenting the samples were different (olfactometer versus jars), with stimulus decay potentially being facilitated by the olfactometer's repeated air flow. Since olfactometers have been the most common way of presenting body odours in the field of chemical communication of emotions [12], our findings regarding stimulus decay are of methodological importance, especially since researchers have more than once reused sweat stimuli [12] up to four times in multi-trial experiments [44], which could have lowered these studies' effect sizes as our study suggests.

Another constraint on generality is that, although our experiment involved a PID to quantify volatiles emitted by sweat, sweat interacts with skin bacteria to form odorous volatiles; yet, the actual composition and abundance of these bacteria were not charted here. Research has indicated that deodorant and anti-perspirant use affects the skin microbiome (e.g. by inhibiting bacterial growth) [45,46], and despite using a standardized ‘washout’ protocol prior to sweat sampling that involved the same scent-free deodorant [8,10,26,33], we cannot rule out variability in composition and abundance of bacteria in donors' armpits and remain unaware of its effects on the production of odorous volatiles. Knowing that different products (deodorants, anti-perspirants) can affect the skin microbiome, future research could address the open yet complex issue of how different bacteria in different quantities interact with sweat produced during particular (intense) states to form volatile molecules.

What also went beyond the scope of this extensive fear encoding study is testing whether *objective* volatile quantity of fear sweat translates into *subjective perception* of its intensity and pleasantness. Importantly, to establish actual chemical communication of fear intensity to a decoder, the next step is to assess whether fear intensity as encoded in the sweat can also be decoded, using the typical non-interfering, implicit measures of behaviour. Such an experiment designed to (repeatedly) measure a biological response to an organism's odour is called a bioassay, which forms an essential part of pheromone identification [47]. Another necessity [47] is then to characterize molecule quality (actual molecule identities) aside from quantity, to test if the same doses of fear sweat and neutral sweat are qualitatively different, and whether higher doses of fear sweat are represented by ‘more of the same’ molecules that would also be found in lower doses of fear sweat. If such olfactory signatures of fear (degree) exist [48], how consistent are they across circumstances, and how broadly are these signatures endorsed by the human species (e.g. across gender, cultures, lifespan)? These constitute important follow-up steps extending the current research.

In the present study, we quantified fear experience by mapping relations between different physiological and experiential response patterns, which has been a fundamental issue since 1884 when William James first conceptualized emotions as distinct coordinated systems that serve adaptive functions [49]. In unpacking the complex relations between experience and physiology, multivariate classification approaches have distinct advantages of univariate ones (e.g. [15,50]). To this end, we used PLS-DA to quantify fear experience (intensity) based on multiple response variables (i.e. HR, GSR, RR, produced armpit sweat and experiential ratings). Our list of parameters was extensive but not exhaustive, lacking for instance neuroendocrine stress markers like cortisol (*slow* stress system product) and salivary α-amylase (*fast* stress system product) [51]. Notably, whereas cortisol has often been collected to determine successful fear induction in sweat donors, α-amylase was shown to be better in distinguishing between video-based fear induction and neutral emotion induction [52]. Indeed, fear sweat production was adaptively tied to fast (not slow) stress system activity [53], with the released adrenalin activating apocrine sweat glands in the armpit [28].

Arguably, fear sweat is more than just ‘arousal sweat’. Our experiential ratings showed patterns of fear, tension and nervousness above and beyond other high arousal–negative affect states. Still, our design was limited by not manipulating other high arousal emotions beyond fear (but see [11]), which could explain the high discriminative potential of our model (100%: fear versus neutral) compared with other multivariate approaches separating *multiple* emotions that share more properties (e.g. fear and anger may both increase HR). Furthermore, if multivariate patterning approaches were based solely on ANS responses, performance decreased (e.g. 44.6% [15], 66.5% [54]), whereas *pairing* physiological and subjective responses caused even anger and fear to be highly separable (99% [54]). Based on this, we currently see no reason to assume that our multivariate approach (which also combined ANS responses and experiential ratings) cannot be used to separate other high arousal emotions (from each other).

By focusing on whether fear intensity could be expressed in the sweat of an *encoder* (apart from its communication to a decoder), our study follows a functional perspective on emotions [21,22], from which it has been argued that emotional expressions primarily have a *self-serving* function by preparing the encoder for perception and action. Specifically, a fearful facial expression has a physical sensory gating function: by opening the eyes, nose and mouth, a greater dose of sensory information (visual and chemical) can be acquired to better deal with threat [21,22]. Arguably, the more ubiquitous and automatic emotion processes become, the more they support the universalist claim that emotions are ‘natural kinds’ with objectively identifiable boundaries (e.g. anger or fear, as opposed to general high arousal–negative affect) that exist across cultures [23]. Whereas emotional facial expressions are determined relatively automatically by the underlying emotion and its intensity [55], they can also be controlled voluntarily, and their presence and degree (like sounds) depend on the social context. Being impervious to these effects, human body odour may form a promising medium to discover ‘natural kind’ fear; encoding of fear intensity in a person's body odour is hard to naturally conceal, which in high fear responders could create a reinforcing cycle of vigilance that may culminate in clinical conditions (e.g. social phobia).

In contrast to this natural kind view on fear, *constructionist* models (e.g. the conceptual-act model) conceptualize emotions as emerging from an interplay between (i) core affect (i.e. valence and arousal) and (ii) accessible conceptual knowledge of emotions [56]. Our findings do not necessarily oppose this model. To chart subjective emotion experience in a theoretically unbiased way, we supplemented items from a dimensional model of affect [36] with non-dimensional, discrete terms (e.g. fear, anger). Whereas the dimensional model indicated that our fear-induced participants experienced high arousal and negative affect (core affect), participants may have narrowed down this core affect experience to ‘fear’ through the accessibility of conceptual knowledge about fear, owing to the present study's clear-cut fear-induction context (scary videos). Following a constructionist perspective, one would then predict that changing the experimental context (e.g. removing conceptual knowledge about fear, or replacing it with anger/disgust) would alter emotion experience [56], a topic that warrants further scrutiny.

## 5. Conclusion

Humans have an excellent sense of smell serving various functions including social communication. Our body odours are a chemical medium conveying our identity, health status and emotions [7,8]. Within the realm of emotion expression, our study suggests that besides emotion quality, its quantity can be encoded in body odour. Such dose–response relations were observed for visual [13] and acoustic [14] modalities, but had been neglected for olfaction. The present research took a novel multivariate approach towards quantifying fear experience intensity and its encoding into volatile molecule quantity. Our research opens new avenues for extending dose–response relations by focusing on decoders and other states/traits deemed relevant for chemical communication.

## Supplementary Material

Supplementary results

## Data accessibility

Data and materials are permanently stored on the Open Science Framework: osf.io/4dtqb/.
