# Supplementary material for: Encoding fear intensity in human sweat
Source: Philos Trans R Soc Lond B Biol Sci. 2020 Apr 20;375(1800):20190271. doi: 10.1098/rstb.2019.0271 (PMC7209933; doi:10.1098/rstb.2019.0271)
Supplement: Supplementary results [file rstb20190271supp1.pdf]

PHILOSOPHICAL
TRANSACTIONS B

Phil. Trans. R. Soc. B.
doi:10.1098/not yet assigned

Supplementary material

Encoding fear intensity in human sweat

Jasper H. B. de Groot[1,2], Peter A. Kirk[1], Jay A. Gottfried[1,3]

[1]Department of Neurology, University of Pennsylvania, 3400 Spruce Street, Philadelphia, PA 19104, USA; [2]Department of Psychology, Utrecht University, Heidelberglaan 1, Utrecht, 3584 CS, the Netherlands; [3]Department of Psychology, University of Pennsylvania, 3720 Walnut Street, Philadelphia, PA 19104, USA

Pilot test: Selecting scary videos to induce fear

Inclusion criteria and test settings (dark room, temperature: 24°C) matched those of the final study. Fifteen males (age: M = 20.33 years, range = 18-22) watched thirteen randomly presented horror clips (65 min) and after each clip, they rated their feelings in return for course credit. Their scores on ten items (0 = "absent"; 5 = "extreme") were averaged into a single discrete *fear spectrum score*, with target states "fearful", "nervous", "tense" keeping their original ratings, and "disgusted", "happy", "angry", "sad", "calm", and "relaxed" being reverse scored. The six clips (~30 min) receiving the highest fear spectrum scores were used in the final study. On average, the chosen film clips (Figure S1) had fear spectrum scores that were significantly (M = 1.91 points) higher than 0, $t(14) = 6.19$, $p < .001$. This highlights the effective induction of a discrete fear-like experience, which varied in strength across subjects (SD = 1.20). Marked variability in fear experiences actually aids the final study's division of subjects into different fear intensity groups (low, medium, and high).

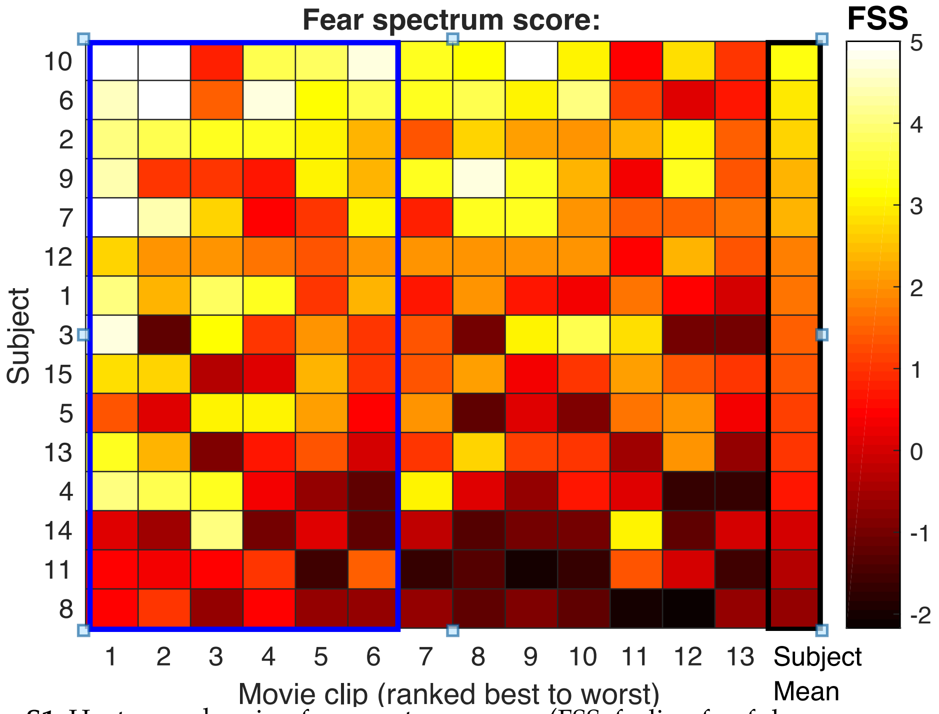

Figure S1. Heat map showing fear spectrum scores (FSS: feeling fearful, nervous, and tense vs. other states) across subjects across film clips (ranked from highest to lowest mean fear spectrum score). Blue rectangle (left): six highest ranked film clips were selected for the final study (~30 min of material). Black rectangle (right): Average FSS per subject showing broad spectrum of subjective fear experience across subjects. Clip #1: "Vicious", 2: "Cop Cam", 3: "100% Organic", 4: "Mr. Creak", 5: "Night Night Nancy", 6: "Blair Witch Project", 7: "The Binding Box", 8: "Rake", 9: "Occupied", 10: "Hostel", 11: "Scream 1", 12: "Copycat".

*Author for correspondence (j.h.b.degroot@uu.nl).
†Present address: Department of Psychology, Utrecht University, Heidelberglaan 1, Utrecht, 3584 CS, the Netherlands

Supplementary Table S1

Descriptive statistics and non-parametric univariate tests comparing responses in the fear condition (to neutral)

	Fear condition<br>*Mdn*<br>(*IQR*)	Neutral condition<br>*Mdn*<br>(*IQR*)	Test
Physiology			
Tonic skin conductance level (μS)	1.72 μS<br>(0.77 - 2.63 μS)	-0.15 μS<br>(-0.94 - 0.48 μS)	$Z = 5.04$, $p < 0.001$, $r = 0.84$
Phasic skin conductance responses (SCR/min)	5.45<br>(3.71 - 8.14)	2.20<br>(1.17 - 4.48)	$Z = 4.85$, $p < 0.001$, $r = 0.81$
Armpit sweat (mg)	175 mg<br>(85 - 570 mg)	95 mg<br>(40 - 197.5 mg)	$Z = 4.12$, $p < 0.001$, $r = 0.68$
Respiratory rate (breaths/min)	17.20<br>(15.87 - 18.53)	16.45<br>(15.34 - 18.05)	$Z = 3.82$, $p < 0.001$, $r = 0.64$
Heart rate (beats/min)	74.15<br>(69.74 - 83.72)	71.83<br>(64.86 - 78.71)	$Z = 2.84$, $p = 0.004$, $r = 0.47$
Self-report ratings: Quadrants			
Low arousal, positive affect (LA+)	0.00<br>(0.00 - 0.00)	3.00<br>(2.60 - 3.90)	$Z = -5.24$, $p < 0.001$, $r = 0.87$
High arousal, negative affect (HA-)	2.44<br>(1.88 - 3.09)	0.00<br>(0.00 - 0.25)	$Z = 5.23$, $p < 0.001$, $r = 0.87$
High arousal, positive affect (HA+)	1.50<br>(0.75 - 2.33)	0.00<br>(0.00 - 1.00)	$Z = 4.03$, $p < 0.001$, $r = 0.67$
Low arousal, negative affect (LA-)	0.00<br>(0.00 - 0.25)	0.13<br>(0.00 - 0.75)	$Z = -2.31$, $p = 0.021$, $r = 0.38$
Self-report: High arousal, negative valence items	Fear condition		Test
Fear (target state)	3.00 (3.00 - 4.00)		
Anger	0.00 (0.00 - 0.00)		$Z = 5.16$, $p < 0.001$ (vs. fear)
Upset	0.00 (0.00 - 2.00)		$Z = 5.12$, $p < 0.001$ (vs. fear)
Disgust	1.00 (0.00 - 3.00)		$Z = 4.25$, $p < 0.001$ (vs. fear)
Stressed	3.00 (1.25 - 4.00)		$Z = 2.13$, $p = 0.033$ (vs. fear)
Nervous	3.00 (3.00 - 4.00)		$Z = -0.85$, $p = 0.397$ (vs. fear)
Tense	4.00 (3.00 - 4.00)		$Z = -1.31$, $p = 0.191$ (vs. fear)
Alert	4.00 (4.00 - 5.00)		$Z = -3.60$, $p < 0.001$ (vs. fear)

Univariate approach: Additional indicators of discrete fear

Wilcoxon signed ranks tests were performed to rule out that fear-induced donors experienced more arousal irrespective of valence (positive vs. negative affect). Indeed, whereas the fear condition yielded the highest ratings on the *HA- item cluster* (e.g., fearful, tense, stressed) versus the other quadrants (HA+, LA-, LA+; *Z*s > 3.65, *ps* < 0.001), the neutral condition evoked the highest scores on the opposing *LA+ item cluster* (e.g., calm, serene), (vs. HA-, HA+, LA-: *Z*s > 5.49, *ps* < 0.001). Overall, the fear condition induced more fear-related feelings (alert, fearful, nervous, and tense) than other non-targeted HA- discrete feeling states (i.e., anger, disgust) (see Table S1, Figure S2).

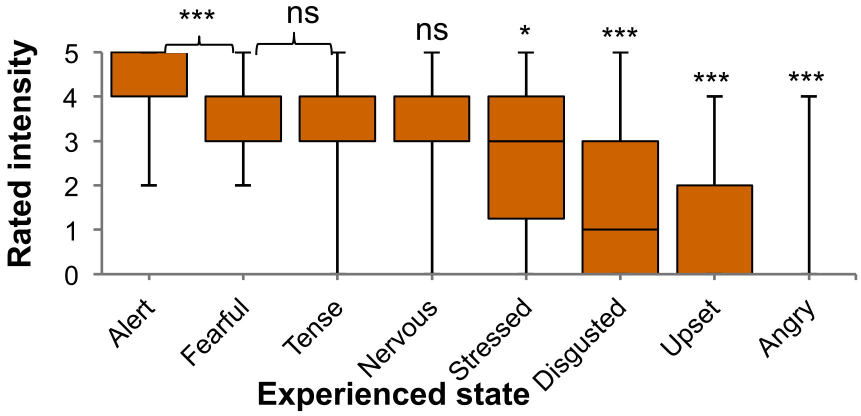

Figure S2. Graph showing fear-induced donors' intensity of experienced feelings on the high arousal, negative affect (HA-) quadrant. Significance values indicate comparisons of that state with the target state "fearful" (ns = not significant; *$p < 0.05$; **$p < 0.01$; ***$p < 0.001$).

Multivariate approach: Relative contribution independent variables to fear-neutral classification
We explored the relative contribution of each subjective and physiological variable in predicting fear class membership as indicated by *standardized* regression coefficients (for an absolute ranking, cf. Figure 3B in the main text). This approach revealed that fear responses were best classified by *low* values on questions such as calmness (LA+ cluster: -0.27) and *high* values on questions such as fear/tension/stress (HA- cluster: 0.26), followed by high galvanic skin responses (SCL: 0.18, SCR: 0.14), higher feelings on the HA+ cluster (0.13), more armpit sweat (0.11), lower feelings on the LA- cluster (-0.08), higher respiratory rate (0.05), and higher heart rate (0.04). The relatively low RR and HR coefficients may be due to more noise (e.g., a finger pulse electrode measured HR, and movements produced artefacts). The relatively high values on self-report measures could be explained by the emotion manipulation (fear, calm-neutral) being obvious and open to reflection.
